# Recurrent Cerebral Embolism Caused by Sluggish Retrograde Flow from the Residual False Lumen of the Aortic Arch after Type A Aortic Dissection

**DOI:** 10.5761/atcs.cr.26-00149

**Published:** 2026-08-07

**Authors:** Norihiro Okada, Shinichiro Shimura, Go Hashimoto, Satoshi Fujita, Morito Hayashi, Hidehiko Hara, Tomohiro Mizuno

**Affiliations:** 1 Department of Cardiovascular Surgery, Toho University Ohashi Medical Center, Tokyo, Japan; 2 Division of Cardiovascular Medicine, Toho University Ohashi Medical Center, Tokyo, Japan; 3 Department of Neurosurgery, Toho University Ohashi Medical Center, Tokyo, Japan

**Keywords:** aortic dissection, false lumen, cerebral embolism, transesophageal echocardiography, four-dimensional computed tomography

## Abstract

A residual false lumen after type A aortic dissection repair may rarely serve as an embolic route to the cerebral circulation. A woman in her 60s underwent ascending aortic replacement for Stanford type A aortic dissection, leaving a residual patent dissection involving the supra-aortic branches and the descending aorta. Despite receiving anticoagulant and antiplatelet therapies, the patient developed recurrent multifocal cerebral infarctions. Transesophageal echocardiography (TEE) demonstrated an entry tear in the descending aorta communicating from the true lumen to the false lumen. Flow became markedly stagnant toward the proximal false lumen, with thrombus-like echogenic material. Four-dimensional computed tomography (4DCT) confirmed delayed retrograde flow ascending within the false lumen toward the arch. Total arch replacement was performed to eliminate the suspected embolic route. Intraoperative findings confirmed thrombotic material within the false lumen. No recurrent cerebral infarctions were observed postoperatively.

## Introduction

Residual aortic dissection after replacement of the ascending aorta for acute type A aortic dissection is usually regarded as being closely associated with late aneurysmal enlargement, rupture risk, and the need for reintervention.^1)^ However, a residual false lumen may create complex flow pathways that promote blood stagnation and thrombogenesis. When such a false lumen communicates with the supra-aortic circulation, it may be a potential source of recurrent cerebral embolism.^2)^ This mechanism is difficult to prove because cerebral embolic events are often multifactorial, and embolic material is rarely identified directly. Nevertheless, multimodal imaging can demonstrate the anatomical and hemodynamic conditions supporting this mechanism: an entry tear, true-to-false-lumen communication, stagnant false-lumen flow with thrombus-prone material, and retrograde flow toward the aortic arch and cerebral circulation. We report the case of a patient with recurrent multifocal cerebral infarctions after type A aortic dissection repair in whom transesophageal echocardiography (TEE) and four-dimensional computed tomography (4DCT) identified a residual arch-descending aortic false-lumen pathway. Surgical closure of the arch false lumen was performed to eliminate the suspected embolic route.

## Case Report

In 2011, a woman in her 60s underwent replacement of the ascending aorta for acute type A aortic dissection. Residual patent dissection remained in the supra-aortic branches and descending thoracic aorta. Her medical history included recurrent cerebral infarctions despite receiving antithrombotic therapy. The patient was treated with warfarin and antiplatelet therapy, which was changed from aspirin to a P2Y12 inhibitor during the clinical course. Electrocardiography revealed sinus rhythm. She developed multifocal cerebral infarctions, predominantly in the right cerebral hemisphere, in 2015, 2018, and 2019. In June 2024, the patient was transported to our hospital with generalized tonic convulsions and impaired consciousness. Brain imaging revealed recurrent multifocal cerebral infarctions in both cerebral hemispheres. No paroxysmal atrial fibrillation was detected during hospitalization.

**Fig. 1:**
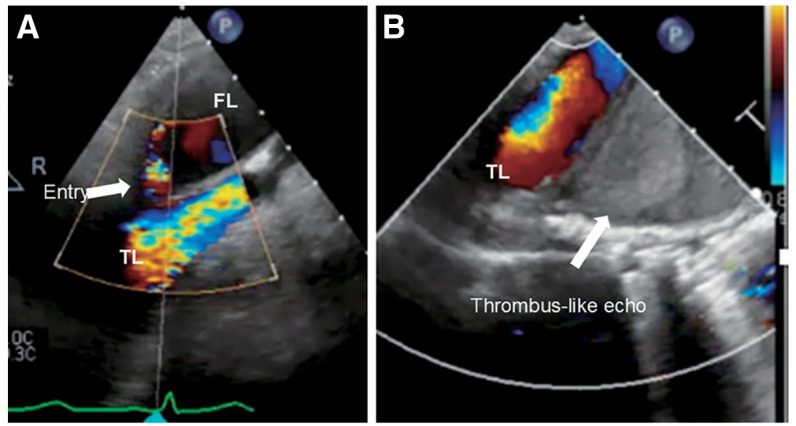
TEE findings. (**A**) Color Doppler imaging demonstrated an entry tear in the descending thoracic aorta, with flow communication from the TL to the FL. (**B**) When the FL was traced proximally toward the aortic arch, flow became markedly stagnant, with spontaneous echo contrast and thrombus-like echogenic material. TL, true lumen; FL, false lumen; TEE, transesophageal echocardiography

Carotid ultrasonography revealed residual dissection of both carotid arteries and abnormal flow within the false lumen. Although the study suggested blood stagnation and communication within the dissected supra-aortic branches, it did not directly establish an embolic pathway from the false lumen to the cerebral circulation. Therefore, TEE was performed to evaluate the thoracic aorta and residual false lumen. It revealed an entry tear in the descending thoracic aorta with flow communication from the true lumen to the false lumen (**Fig. 1A**). When the false lumen was traced proximally toward the aortic arch, the flow became progressively slower and more stagnant, accompanied by spontaneous echo contrast and thrombus-like echogenic material (**Fig. 1B**). These findings suggest that the blood entering the residual false lumen through the descending aortic entry tear becomes increasingly stagnant within the proximal false lumen, creating a thrombogenic environment.

**Fig. 2:**
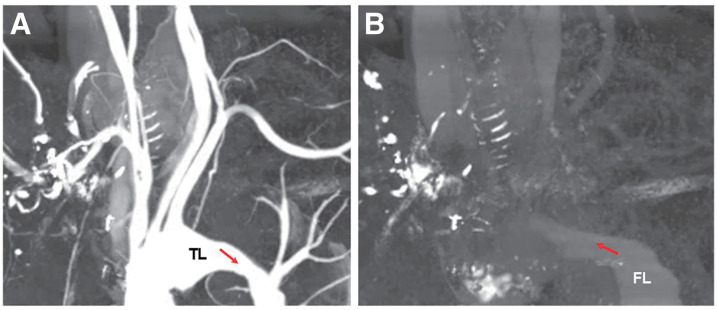
4DCT findings. (**A**) Early-phase images demonstrated antegrade opacification predominantly within the TL. (**B**) Delayed-phase images demonstrated retrograde opacification ascending within the FL toward the aortic arch, indicating that the residual FL was not an isolated thrombosed cavity but rather a delayed-flow pathway communicating with the proximal aorta and supra-aortic circulation. TL, true lumen; FL, false lumen; 4DCT, four-dimensional computed tomography

4DCT further clarified the global hemodynamic pathway. Early-phase images showed antegrade opacification predominantly within the true lumen, whereas delayed-phase images demonstrated retrograde opacification ascending within the false lumen toward the aortic arch, and the residual arch false lumen communicating with the supra-aortic circulation (**Figs. 2A** and **2B**, **Supplementary Video 1**). Based on these findings, the most plausible mechanism for recurrent cerebral emboli is retrograde sluggish blood flow into the supra-aortic vessels from the residual false lumen of the aortic arch and descending aorta. Therefore, surgery was planned to eliminate the suspected embolic route.

**Fig. 3:**
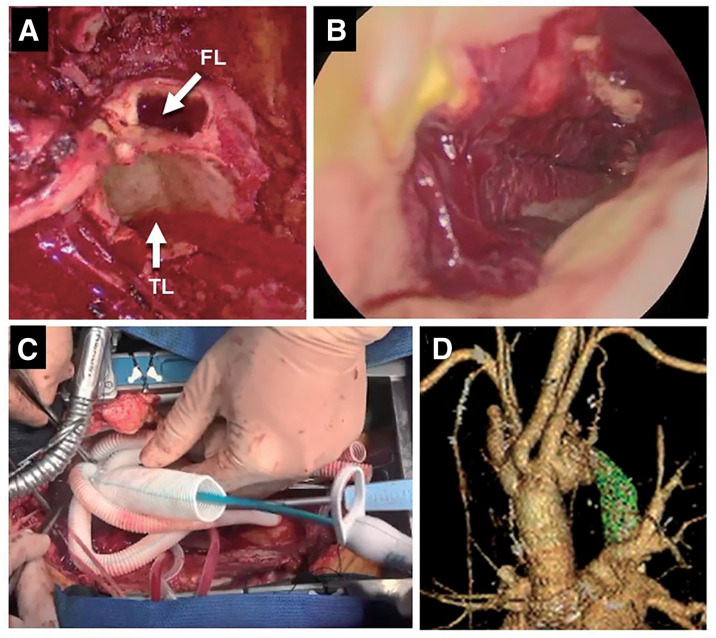
Operative findings and postoperative CT. (**A**) The TL and FL were identified at the zone 2 level. (**B**) Intraoperative endoscopic observation demonstrated thrombotic material within the FL. (**C**) The distal open stent graft component of the Frozenix 4-branched frozen elephant trunk graft was deployed to expand the TL and exclude the FL pathway. (**D**) Postoperative 3-dimensional CT demonstrated replacement of the aortic arch, with exclusion of the target arch FL communication. TL, true lumen; FL, false lumen; CT, computed tomography

Redo median sternotomy was performed, and the dissected aortic arch was replaced with a 24-mm × 25-mm × 9-cm Frozenix 4-branched frozen elephant trunk graft (Japan Lifeline Co., Ltd., Tokyo, Japan) using selective antegrade cerebral perfusion and lower body circulatory arrest at 26°C. Intraoperative endoscopy revealed a fresh thrombus within the false lumen, extending from the aortic arch to the descending aorta (**Figs. 3A** and **3B**). As a result, the residual arch false lumen containing fresh thrombotic material was excluded from the supra-aortic circulation using the frozen elephant trunk graft (**Fig. 3C**). The postoperative course was uneventful. Postoperative 3-dimensional CT demonstrated replacement of the aortic arch with exclusion of the target arch false-lumen communication (**Fig. 3D**). The patient remained ambulatory without recurrent cerebral infarction during the 2-year postoperative follow-up period.

## Discussion

This case illustrates a rare but clinically important mechanism of recurrent cerebral embolism after the repair of a type A aortic dissection. The distinctive feature was not merely the presence of residual dissection or false-lumen thrombus. Rather, a sequential embolic pathway was demonstrated: true-lumen inflow into the descending aortic false lumen through an entry tear, progressive stagnation and thrombus-like material within the proximal false lumen, delayed retrograde flow toward the arch, and communication between the arch false lumen and the supra-aortic circulation. This configuration provides a plausible route for thrombotic material generated in the residual false lumen to embolize retrogradely toward the cerebral circulation.

Neurological complications of acute type A aortic dissection and postoperative residual branch-vessel dissection have been recognized, and possible mechanisms include hemodynamic hypoperfusion, extension of the dissection into cerebral vessels, and artery-to-artery embolism.^2)^ However, in patients with recurrent stroke after aortic dissection repair, the underlying mechanism is often difficult to identify. Cardioembolic sources, atherosclerotic embolism, carotid artery disease, and hemodynamic compromise should also be considered. In the present patient, repeated cerebral infarctions occurred despite anticoagulant and antiplatelet therapy. No paroxysmal atrial fibrillation was detected, and imaging identified a residual false-lumen pathway that communicated with the cerebral circulation. Although direct proof that a specific thrombus fragment migrated into the cerebral arteries was not possible, the anatomical and hemodynamic findings strongly support residual arch false-lumen communication as the most plausible treatable source.

TEE is central to identifying the local mechanism. Carotid ultrasonography can demonstrate residual branch dissection and abnormal false lumen flow, but the thoracic false-lumen pathway cannot be fully evaluated. TEE directly visualizes the descending aortic entry tear and true-to-false-lumen communication. It also shows that the proximal false lumen has markedly stagnant flow with spontaneous echo contrast and thrombus-like material, indicating a thrombus-prone environment.^3,4)^ This real-time assessment was important because static CT may depict the anatomy but may not fully characterize sluggish flow and spontaneous echo contrast.

4DCT complemented echocardiography by demonstrating the global direction of flow within the residual dissected aorta. Delayed-phase retrograde opacification ascending in the false lumen toward the arch suggested that the thrombogenic false-lumen compartment was not isolated. Instead, it was part of a pathway capable of delivering material toward the supra-aortic circulation. Therefore, the combination of echocardiography and 4DCT strengthens the rationale for treatment directed at the embolic route, rather than further escalation of systemic antithrombotic therapy.^5)^

To the best of our knowledge, no previous report has specifically demonstrated recurrent cerebral embolism caused by retrograde migration of thrombotic material through a residual aortic false lumen communicating with the supra-aortic circulation after repair of acute type A aortic dissection. This case suggests that, in patients with recurrent cerebral infarction after replacement of the ascending aorta for acute type A aortic dissection, clinicians should actively evaluate whether residual false-lumen communication provides a treatable route for retrograde thromboembolism.

The operative strategy was not simply to reconstruct the aorta, but to close the residual arch false lumen that communicated with the supra-aortic circulation and remove thrombotic material from the false lumen. The intraoperative finding of fresh thrombotic material within the false lumen supports the preoperative interpretation that the false lumen was thrombogenic. Since the embolic route is anatomically defined and surgically addressable, closure of the arch false lumen by total arch replacement is considered a rational therapeutic strategy. The absence of recurrent cerebral infarction after surgery further supports the clinical relevance of this approach, although a longer follow-up period is required.

## Conclusion

A residual arch-descending aortic false lumen after replacement of the ascending aorta for acute type A aortic dissection may serve as a source of recurrent cerebral embolism when the false lumen of the aortic arch communicates with the supra-aortic circulation. TEE and 4DCT were complementary in demonstrating true-to-false-lumen communication, stagnant thrombus-prone flow, and delayed retrograde flow toward the arch.

## Supplementary Materials

Supplementary Video 1 Four-dimensional computed tomography findings. The early-phase sequence shows predominant antegrade opacification of the true lumen, whereas the delayed-phase sequence reveals retrograde opacification of the false lumen toward the aortic arch.

## Declarations

### Use of artificial intelligence tools

Artificial intelligence-assisted tools were used only for English language editing, and the manuscript was revised by a native English speaker before submission. No artificial intelligence tools were used for data collection, data analysis, image interpretation, clinical decision-making, generation of scientific content, or drawing conclusions. All authors critically reviewed and edited the manuscript and remain fully responsible for its accuracy, integrity, and final content.

### Availability of data and materials

The data are available from the corresponding author upon reasonable request.
